# Physical Operations of a Self-Powered IZTO/β-Ga_2_O_3_ Schottky Barrier Diode Photodetector

**DOI:** 10.3390/nano12071061

**Published:** 2022-03-24

**Authors:** Madani Labed, Hojoong Kim, Joon Hui Park, Mohamed Labed, Afak Meftah, Nouredine Sengouga, You Seung Rim

**Affiliations:** 1 Laboratory of Semiconducting and Metallic Materials (LMSM), University of Biskra, Biskra 07000, Algeria; madani.labed@univ-biskra.dz (M.L.); af.meftah@univ-biskra.dz (A.M.); n.sengouga@univ-biskra.dz (N.S.); 2 Department of Intelligent Mechatronics Engineering, and Convergence Engineering for Intelligent Drone, Sejong University, Seoul 05006, Korea; hkim3023@gatech.edu (H.K.); julia980406@gmail.com (J.H.P.); 3 George W. Woodruff School of Mechanical Engineering, Institute for Electronics and Nanotechnology, Georgia Institute of Technology, Atlanta, GA 30332, USA; 4 High Collage of Food Sciences and Food Industries, Algiers 16200, Algeria; labed@essaia.dz

**Keywords:** IZTO/β-Ga_2_O_3_ Schottky diode, solar-blind, self-powered, photodetector, modeling

## Abstract

In this work, a self-powered, solar-blind photodetector, based on InZnSnO (IZTO) as a Schottky contact, was deposited on the top of Si-doped β-Ga_2_O_3_ by the sputtering of two-faced targets with InSnO (ITO) as an ohmic contact. A detailed numerical simulation was performed by using the measured J–V characteristics of IZTO/β-Ga_2_O_3_ Schottky barrier diodes (SBDs) in the dark. Good agreement between the simulation and the measurement was achieved by studying the effect of the IZTO workfunction, β-Ga_2_O_3_ interfacial layer (IL) electron affinity, and the concentrations of interfacial traps. The IZTO/β-Ga_2_O_3_ (SBDs) was tested at a wavelength of 255 nm with the photo power density of 1 mW/cm^2^. A high photo-to-dark current ratio of $3.70\times 10^{5}$ and a photoresponsivity of 0.64 mA/W were obtained at 0 V as self-powered operation. Finally, with increasing power density the photocurrent increased, and a 17.80 mA/W responsivity under 10 mW/cm^2^ was obtained.

## 1. Introduction

Gallium oxide (Ga_2_O_3_) is an oxide semiconductor material with a long, rich history [1,2,3]. It has an ultra-wide bandgap (UWBG) of ~4.8 eV, a high breakdown electric field of ~8 MV/cm, and a high saturation velocity of 1 × 10^7^ cm/s, and these properties have brought Ga_2_O_3_ to the fore once again [1,2,4]. Ga_2_O_3_ has six polymorphs, i.e., α, β, γ, δ, ε, and k, with β-Ga_2_O_3_ being the most stable [1]. Unipolar devices based on β-Ga_2_O_3_, such as the metal–oxide–semiconductor field-effect transistor (MOSFET) [5], thin film transistor (TFT) [6], field emission (FE) [7], and Schottky barrier diode (SBD) [1,2,3,4,8,9], have been studied extensively. It is also used for deep ultraviolet (DUV) photodetectors (PDs) for solar-blind applications [10,11]. DUV PDs work in the solar-blind spectrum with wavelengths shorter than 280 nm, which means that they can be applied to optical communication, chemical analysis, missile tracking, and harsh environmental monitoring [12]. Different types of solar-blind PD structures based on β-Ga_2_O_3_, such as metal–semiconductor–metal [13], heterojunction [14], and Schottky [15], have been reported. The Schottky barrier diode solar-blind has some advantages, including low dark current and low cost in comparison with heterojunctions [11,16]. Self-powered solar-blind PDs are of special interest, because they can work in the absence of an external power supply. A strong built-in electric field ensures that this high-performance, self-powered, solar-blind Schottky barrier diode photodetector can operate at zero bias voltage [14]. Different metals have been used as the Schottky contacts of β-Ga_2_O_3_, e.g., Au, Ti, Ni, Pt, Cu, and Pb [17]. For example, Chen et al. [18] reported a self-powered photodetector based on a Au/β-Ga_2_O_3_ nanowire array film Schottky, in which the responsivity reached 0.01 (mA/W) during 254 nm light illumination with 2 mW/cm^2^ at a bias of 0 V. Zhi et al. [19] studied the Au/β-Ga_2_O_3_ Schottky solar-blind photodetector, and they achieved a responsivity of 0.4 (mA/W) for 0 V bias and 254 nm illumination. Peng et al. [10] reported a Pt/β-Ga_2_O_3_ Schottky barrier diode solar-blind photodetector with nearly a 10^4^ of light to dark current ratio at 0 V bias and a wavelength of 254 nm. Liu et al. [20] studied a Ni/β-Ga_2_O_3_ Schottky barrier diode solar-blind photodetector tested under 254 nm light, and they obtained responsivities of about 806.02 (A/W) and 1372.92 (A/W) under −5 V and 5 V, respectively. In the publications mentioned above, various metals were used, such as Pt, Ni, and Au, for Schottky contact formation with β-Ga_2_O_3_. In addition, transparent materials and oxides are used for the formation of the Schottky contact with β-Ga_2_O_3_. However, these types of materials formed a low Schottky barrier height with β-Ga_2_O_3_. For example, Zhuo et al. [14] reported a MoS_2_/β-Ga_2_O_3_ heterojunction self-powered photodetector with a 670 ratio of light current to dark current at 0 V, and this result was achieved because of the very low value of the Schottky barrier. Chen et al. [17] studied a self-powered MXenes/β-Ga_2_O_3_ photodetector under 254 nm wavelength with a light illumination of 115.1 µW.cm^−2^ and a 1.6 × 10^4^ ratio of light current to dark current at 0 V and an extracted Schottky barrier height of about 0.9 eV. Then, Cui et al. [21] published a flexible solar-blind amorphous β-Ga_2_O_3_ photodetector with an indium tin oxide (ITO) transparent conducting electrode, with a photocurrent that is less effected at 0 V and under 254 nm when it is exposed to an oxygen flux of 0.14 SCCM; this result was related to the low Schottky barrier height of about 0.97 eV. In addition, Kim et al. [11] used InZnSnO (IZTO) for Schottky contact formation with β-Ga_2_O_3_**,** and a Schottky barrier height greater than 1.06 eV was obtained; these results indicate that this photodetector can work in the absence of an external power supply.

Here, we constructed simulation–experiment combination of ITZO/β-Ga_2_O_3_ SBD-based UV photodiodes under the illumination to reveal the physics behind the behavior of the J–V characteristics in terms of workfunction, IL electron affinity and interfacial traps. The different conduction mechanisms we performed were taken into consideration either collectively or individually. Their parameters were scanned over a physically acceptable range so that an acceptable comparison of measurement to simulation was achieved. Good agreement between simulation and experiment was observed clearly with the consideration of the effect of different IZTO workfunctions, IZTO/β-Ga_2_O_3_ interfacial layer electron affinity, and the effect of interfacial traps. Additionally, the IZTO/β-Ga_2_O_3_ self-powered solar-blind PDs were tested and compared with measurements at the photo power density of 1 mW/cm^2^ and the wavelength of 255 nm.

## 2. Experiment

**Figure 1:**
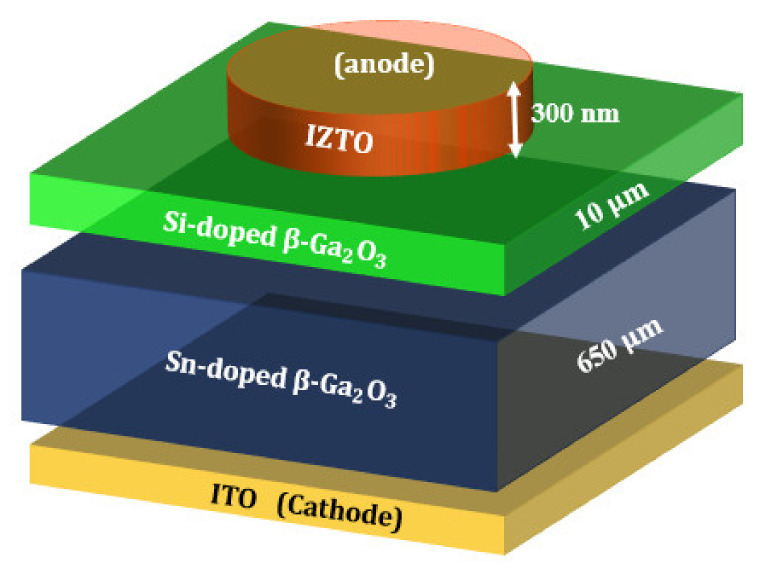
A schematic representation of the β-Ga_2_O_3_ Schottky barrier diode (SBD) structure.

The Solar-blind Schottky photodetector was fabricated on a 650 µm, Sn-doped, bulk β-Ga_2_O_3_ single-crystal wafer ((N_d_−N_a_) = 1 × 10^18^ cm^−3^, Novel Crystal Technology, Inc., Saitama, Japan) with (001) surface orientation. The epitaxial layer of Si-doped β-Ga_2_O_3_ (10 µm thick, 1 × 10^18^ cm^−3^) was grown by halide vapor phase epitaxy (HVPE). Si-doped β-Ga_2_O_3_ was used as the active layer in this solar-blind Schottky photodetector, as it provides a high purity and a low resistance [2]. The ITO electrode was deposited by sputtering on the bottom of Sn-doped β-Ga_2_O_3_ as an ohmic contact. IZTO was deposited on the top of the Si-doped β-Ga_2_O_3_ as a Schottky contact by two-faced target co-sputtering with ITO (In_2_O_3_:SnO = 9:1) and IZO (In_2_O_3_:ZnO = 9:1) at room temperature. Figure 1 shows a schematic representation of this SBD structure. After the deposition of the layers, the device was annealed at 600 °C in Ar for 1 min using rapid thermal annealing. The electrical J–V in dark and light was measured using a semiconductor analyzer and a source meter (SCS-4200A and 2410 Source meter, Keithley, Beaverton, OR, USA). Further details can be found in our previous publication [11].

## 3. Simulation Methodology

For the simulation, we considered thermionic emission, Shockley–Read–Hall, Auger recombination, and image force lowering models. The physical parameters of different layers and related traps are presented in Table 1 and Table 2, respectively.

SILVACO TCAD (Version 5.24.1.R, Silvaco Inc.: Santa Clara, CA, USA) was used to model the above structure. It solves the basic drift–diffusion semiconductor Poisson and continuity equations, which are [2,3,22]:

Poisson equation is given by [2,3,22]:

(1) $$div\left(\varepsilon \nabla \psi \right)=-q\left(p-n+N_{d}\pm N_{t}^{\pm }\right)$$

where ψ is the electrostatic potential, ε is the permittivity, p and n are the concentrations of the free holes and electrons, respectively, and $N_{t}^{\pm }$ is the density of the ionized traps $\left({\mathrm{cm}}^{-3}\right)$.

The continuity equations for electrons and holes as defined in steady states are given by [2,3,22]:

(2) $$0=\frac{1}{q}div\vec{J_{n}}+G_{n}-R_{n}$$

(3) $$0=-\frac{1}{q}div\vec{J_{p}}+G_{p}-R_{p}$$

where $G_{n}$ and $G_{p}$ are the generation rates for electrons and holes, respectively, and $R_{n}$ and $R_{p}$ are the recombination rates for electrons and holes, respectively. ${\vec{J}}_{n}$ and ${\vec{J}}_{p}$ are the electron density and the hole current density, respectively, which are given in terms of the free electron and hole density (n and *p*), electric field (E) and mobility ($\mu_{n}$ and $\mu_{p}$) [25]:

(4) $${\vec{J}}_{n}=q\mu_{n}nE+\mu_{n}K_{B}T\nabla n$$

(5) $${\vec{J}}_{p}=q\mu_{p}pE-\mu_{p}K_{B}T\nabla p$$

Traps are represented by their ionized density, $\;N_{t}^{\pm }$. The sign ± depends on whether the trap is an acceptor or a donor, so that $N_{t}^{+}=fN_{t}$ and $\;N_{t}^{-}=\left(1-f\right)N_{t}$, f is the occupancy function given by $\;f=\frac{\sigma_{n}n+\sigma_{p}p}{\sigma_{n}\left(n+n_{t}\right)+\sigma_{p}\left(p+p_{t}\right)}$, and $\sigma_{n\left(p\right)}$ is the trap capture cross-section for electrons (holes). The recombination rate is related to traps through the well-known SRH formula, i.e., $R_{n,p}=\frac{pn-n_{i}^{2}}{\tau_{0n}\left(p+p_{t}\right)+\tau_{0p}\left(n+n_{t}\right)}$, where $n_{t}=n_{i}exp\left(-\left(E_{i}-E_{t}\right)/kT\right)$ and $\;p_{t}=n_{i}exp\left(-\left(E_{t}-E_{i}\right)/kT\right)$, and $\tau_{0n}$ and $\tau_{0p}$ are the minority carrier lifetimes which are also related to the traps through $\tau_{0n\left(p\right)}=\frac{1}{v_{thn\left(p\right)}\sigma_{n\left(p\right)}N_{t}}$, where $v_{thn\left(p\right)}$ is the thermal velocity of electrons (holes).

According to several publications [11,26,27], ITO forms an Ohmic contact with β-${\mathrm{Ga}}_{2}O_{3}$. In this simulation, an ideal ohmic contact was considered for ITO/β-Ga_2_O_3_ interface. A low ITO/β-Ga_2_O_3_ barrier was achieved when highly doped β-${\mathrm{Ga}}_{2}O_{3}$ substrate was used.

## 4. Results and Discussion

### 4.1. Optical and Electrical Properties of IZTO Thin Film

**Figure 2:**
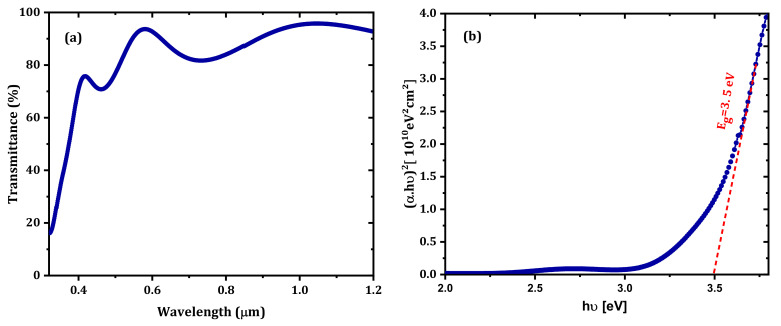
(**a**) The transmittance of IZTO thin film; (**b**) (α (λ).hυ) ^2^ of the IZTO thin film.

The obtained resistivity, carrier concentration, workfunction, and mobility of IZTO were $4.86\times 10^{-4}$ Ω cm, $2.80\times 10^{20}\;{\mathrm{cm}}^{-3}$, 4.79 eV, and 10.83 cm^2^/V s, respectively. Figure 2a shows the optical transmission ($T\left(\lambda \right))$ of IZTO thin film as a function of the wavelength in the 250–1200 nm range. The average transmittance of the IZTO films in the visible wavelength range was over 87%.

**Figure 3:**
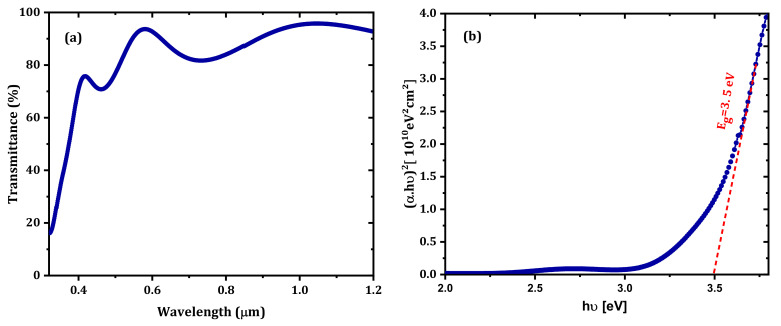
The extracted refractive index (n) and the extinction coefficient (k) of IZTO thin film.

For direct bandgap semiconductors, the absorption can be obtained from the following equation [15]:

(6) $${\left(\alpha h\nu \right)}^{2}=C\cdot \left(h\nu -E_{g}\right)$$

where α is the absorption coefficient, *C* is a constant, *h* is Planck’s constant, and ν is the frequency of the incident light. By plotting (α (λ)·hυ)^2^ versus hυ, the optical bandgap of the IZTO thin film was determined to be 3.5 eV, as shown in Figure 2b. This value agrees with the published value [28]. The refractive index has a significant importance in the design of optical devices. It reflects the crystallinity and optical quality of thin films. The extinction coefficient (k) and the refractive index (n) of IZTO thin film are calculated by [29]:

(7) $$k\left(\lambda \right)=\frac{\alpha .\lambda }{4\pi }$$

(8) $$n\left(\lambda \right)=\frac{\left(1-R\left(\lambda \right)\right)}{\left(1+R\left(\lambda \right)\right)}+\sqrt{\frac{4R\left(\lambda \right)}{1-R{\left(\lambda \right)}^{2}}-k{\left(\lambda \right)}^{2}}$$

where $R\left(\lambda \right)$ is the reflectance of the thin film, which can be calculated by the following equation [30]:

(9) $$R\left(\lambda \right)=1-\sqrt{T\left(\lambda \right)}e^{\frac{\alpha .t}{2}}$$

where t is the IZTO thin film thickness, which is evaluated by ellipsometry at ≈300 nm. The extracted refractive index (n) and the extinction coefficient (k) are presented in Figure 3.

As mentioned above, the photodetector consisted of 300 nm IZTO deposited on the top of Si-doped β-Ga_2_O_3_, and the ITO layer was considered as an ohmic contact on the bottom of the Sn-doped β-Ga_2_O_3_ (Figure 1). Before simulating the proposed photodetector, the measurement of the dark output current density of SBD was reproduced, and details are provided in the next section.

### 4.2. Modeling the Dark Current of IZTO/β-Ga_2_O_3_ SBD

**Figure 4:**
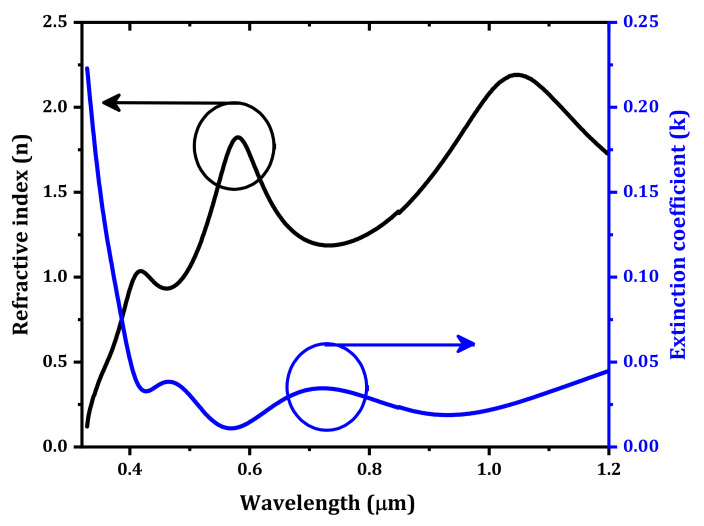
IZTO/β-Ga_2_O_3_ SBD forward current density with and without the tunneling model compared to measurement.

As presented in Figure 4, when the tunneling transport mechanism was not considered, the shape of the IZTO/β-Ga_2_O_3_ SBD current is parallel to the measurement current; this indicates that the thermionic transport mechanism dominates in the forward bias. However, when the properties presented in Table 1 and the traps presented in Table 2 were considered, a huge disagreement was obtained between the simulation and the measurement. This disagreement is related to the IZTO workfunction, i.e., the IZTO/β-Ga_2_O_3_ IL electron affinity (conduction band minimum), in addition to the effect of the surface traps between IZTO and the β-Ga_2_O_3_ drift layer.

### 4.3. Effect of IL Electron Affinity

**Figure 5:**
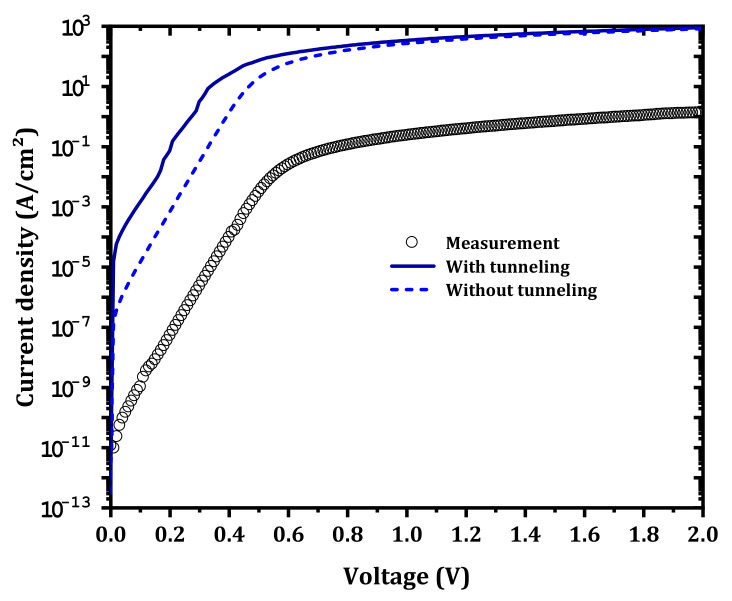
Effect of the IL electron affinity on the simulated J–V characteristics compared to measurement.

**Figure 6:**
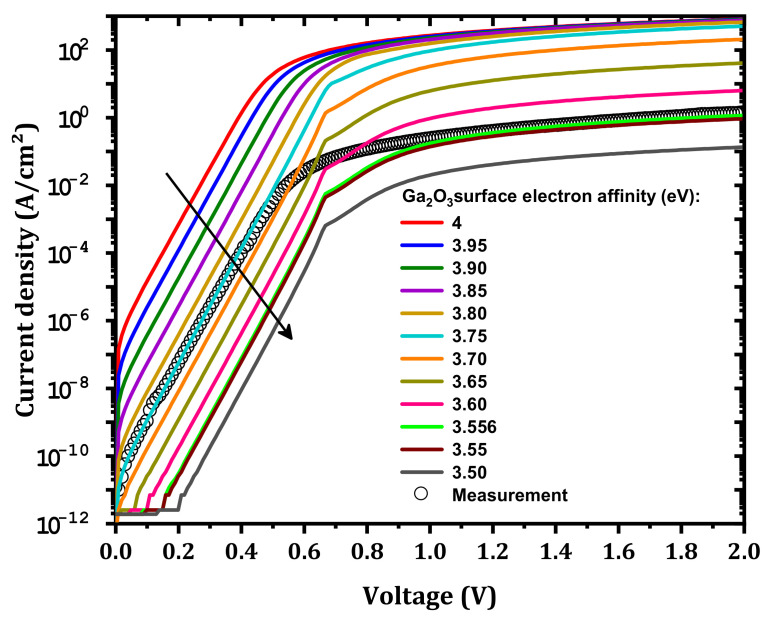
Equilibrium band diagram variation with the IL electron affinity.

Next, an IL (10 nm thickness) between Si-doped β-Ga_2_O_3_ and IZTO was considered for modeling the effect IZTO/β-Ga_2_O_3_ conduction band offset on the SBD performance. The effects of the IL electron affinity on the SBD J–V characteristics were studied, and these effects are shown in Figure 5. Experimentally, the IL electron affinity is related to the chemical composition of the surface [2] and surface polarization [31] as well as external effects, such as argon (Ar) bombardment, plasma, etc. The current density decreases with decreasing IL electron affinity of Si-doped β-Ga_2_O_3_, i.e., from 4 to 3.5 e V [32], but the IL electron affinity has a more pronounced effect in the high voltage domain. This is due to the increase in the height of the Schottky barrier ($\phi_{B}$) with decreasing IL electron affinity according to the Schottky–Mott rule and the increase in the series resistance [2]. As presented in Figure 6, with decreasing IL electron affinity, the barrier between IZTO and β-Ga_2_O_3_ increases. An agreement between measurement and simulation occurs for voltages higher than 1 V for a 3.556 eV IL electron affinity. However, a disagreement between simulation and measurement was noticed in the low-voltage domain, and this is related to the effect of the IZTO workfunction and the concentrations of the interfacial traps, which are addressed in the next two subsections.

### 4.4. Effect of the IZTO Workfunction

**Figure 7:**
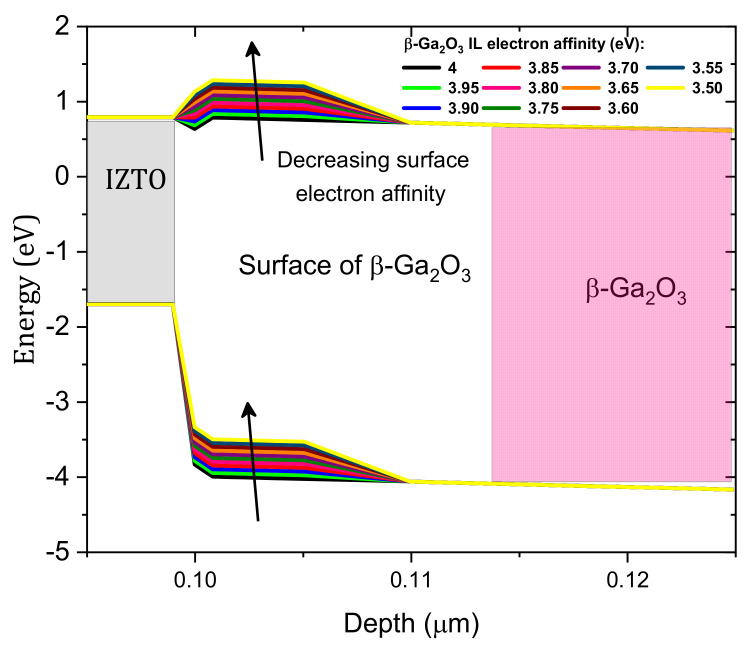
Simulated J–V characteristics for different IZTO workfunctions compared to measurements.

**Figure 8:**
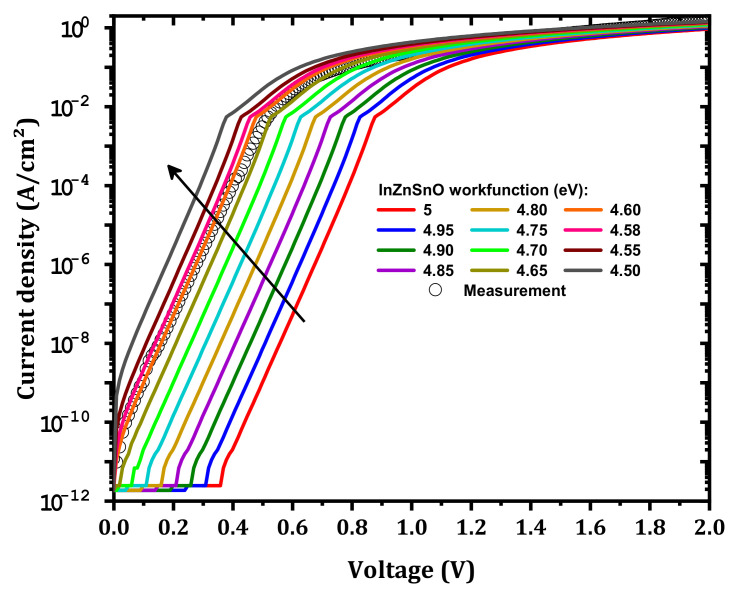
Equilibrium band diagram variation with the IZTO workfunction.

In addition to the IL electron affinity, the IZTO workfunction will have an effect. As shown in Figure 7, when the IZTO workfunction decreases from 5 to 4.5 eV [33,34] the current density increases. This increase in the current density is related to the decrease in $\phi_{B}$ as presented in Figure 8; the formed barrier between IZTO and Si-doped β-Ga_2_O_3_ increased. The best agreement between simulation and measurement was achieved for $\phi_{IZTO}=4.6\;\mathrm{eV}$. The small deviation from measurement, i.e., in the range of 0.4–0.8 V, was due to the parameters of the traps that are considered (Table 2), which may not be accurate. It also may be related to the effect of the different compositions of the materials (In, Zn, and Sn) [35].

### 4.5. Effect of the Concentration of Traps at the IL

**Figure 9:**
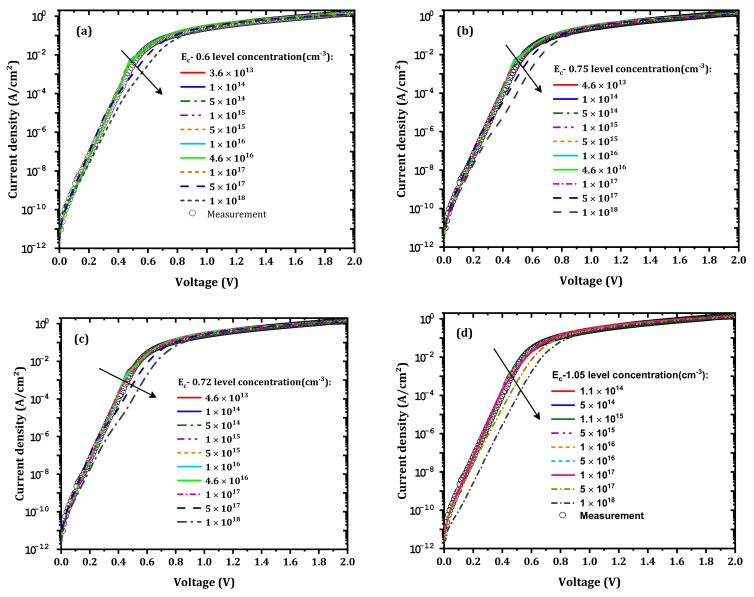
Effects of the density of the traps on SBD, i.e., the (**a**) $E_{c}-0.6\;\mathrm{eV},$ (**b**) $E_{c}-0.75\;\mathrm{eV},$ (**c**) $E_{c}-0.72\;\mathrm{eV},$ and (**d**) $E_{c}-1.05\;\mathrm{eV}$ traps.

**Figure 10:**
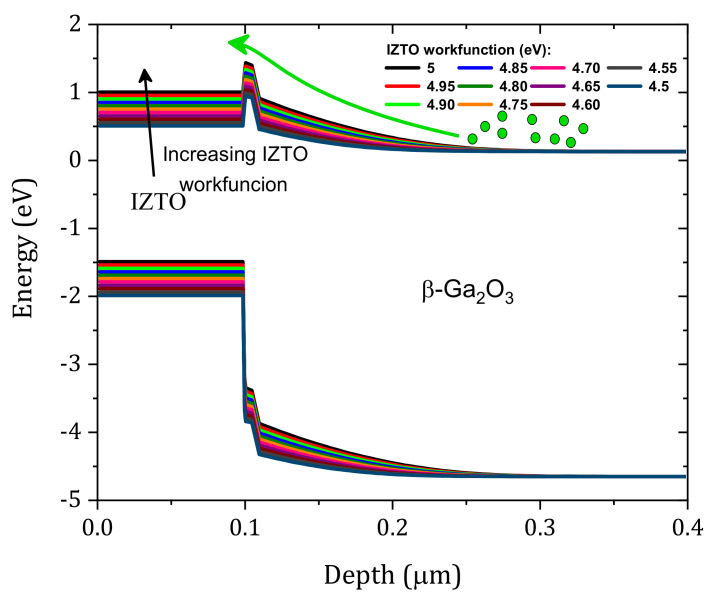
Equilibrium band diagram variation with and without traps.

**Figure 11:**
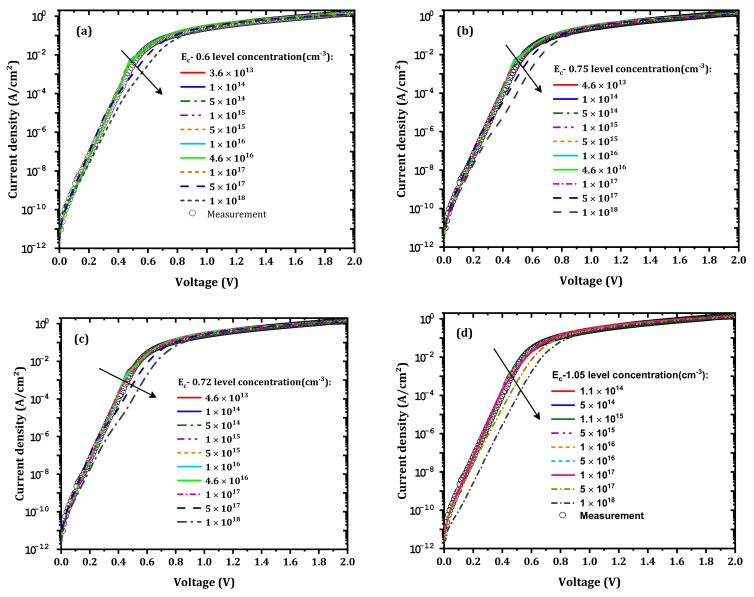
The best comparison between the simulation and the experimental results.

We studied the effect of the concentration of the (E_c_−1.05) traps at IL on the characteristics of the SBD J–V. The traps that we considered were the most affected, especially given that the surface of the β-Ga_2_O_3_ is exposed to plasma and Ar bombardment [2]. For the four traps, these effects are shown in Figure 9a–d, respectively. First, all of the defects that were considered have a significant effect on the current density of the SBD. Among the defect concentrations that were considered, those that gave the best comparison with the measurements were $3.6\times 10^{16}$,$\;4.6\times 10^{16}$, $4.6\times 10^{16}$ and $1.1\times 10^{15}\;{\mathrm{cm}}^{-3},$ respectively. The dark current was affected at high trap densities, and this result was related to electrons being captured by the traps. Figure 10 shows the effect of traps on the equilibrium band diagram. When traps were considered with the concentration mentioned above, the difference of conduction band and Fermi level (E_c_ – E_f_) increased, and this meant a decrease in the free electron density. A good comparison between simulation and measurement is obtained as presented in Figure 11 and the extracted SBD parameters are presented in Table 3. The Schottky barrier height ($\phi_{B}$) and the R_s_ were extracted using the Sato and Yasumura method [3,36]. A high $\phi_{B}$ was obtained with a low ideality factor close to unity in addition to the low densities of the interfacial traps, as expected in our previous publication [11]. In addition, a very low saturation current was obtained.

### 4.6. The Effect of 255 nm Wavelength Illumination on Forward Current

**Figure 12:**
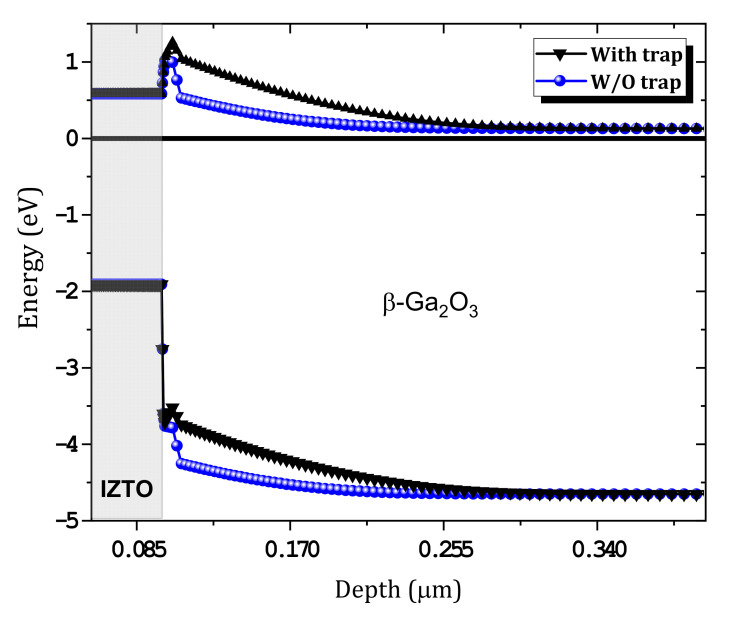
Comparison between simulation and measurement (under incident light power density of 1 mW/cm^2^) of J–V characteristics of IZTO/β-Ga_2_O_3_ at 255 nm wavelength.

The simulated SBD J–V characteristics were successfully compared to measurements made at room temperature. This good agreement was achieved by modeling the effect of IL electron affinity, IZTO workfunction, and the concentrations of the IL traps. As shown in Table 3, a low dark saturation current was obtained, so this SBD is proposed as a high-performance solar-blind PD. The IZTO/β-Ga_2_O_3_ SBD was illuminated at 255 nm with a light intensity of 1mW/cm^2^. To evaluate the performance of the IZTO/β-Ga_2_O_3_ solar-blind SBD under 255 nm, the forward photocurrent was extracted with the consideration of the previous IZTO workfunction, the IL electron affinity, and the concentrations of the interfacial traps. As presented in Figure 12**,** good agreement was demonstrated between the simulation results and the actual measurements. The solar-blind SBD exhibited a high rectifying characteristic after illumination at 255 nm in forward voltage $\left(J_{Photon}\;/\;J_{dark}=3.70\times 10^{5}\right)$ under 0 V. The responsivity reached 0.64 (mA/W). The responsivity was estimated as follows [37]:

(10) $$R_{\lambda }=\frac{J_{Photon}-J_{dark}}{P}\;$$

where $J_{Photon}$, $J_{dark}$, and P are the photocurrent at a given voltage, the dark current, and the power density, respectively. With zero bias voltage, the photodetector had good responsivity under 255 nm light illumination with intensity of 1 (mW/cm^2^). In addition, a decrease in $\phi_{B}$ from 1.25 eV in dark to 1.18 after illumination was observed.

**Figure 13:**
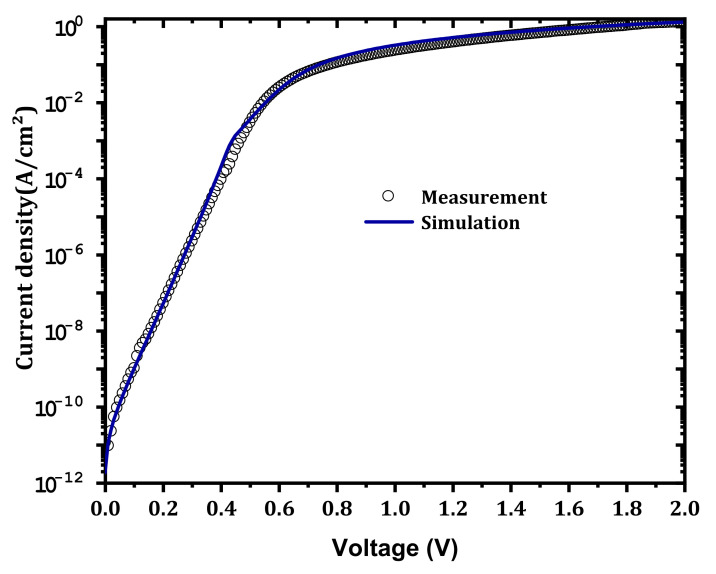
Effect of light power density on the simulated J–V characteristics. The inset is the variation of photocurrent with light power density at 0 V.

In this solar-blind PD, the built-in electric field in the depletion region of the β-Ga_2_O_3_ and IZTO interface was enough to separate the photogenerated electron–hole pairs toward corresponding electrodes. In this structure, a high built-in potential is related to the high $\phi_{B}$. In addition, the effect of light power density on simulated J–V characteristics was studied. As presented in Figure 13, with increasing power density, the photocurrent increased, and a responsivity of 17.80 mA/W under 10 mW/cm^2^ was achieved. This increase in photocurrent is related to the increase in photo-excited, separated, and collected carriers [38]. This result agrees with the result obtained by Wu et al. [39].

## 5. Conclusions

The J–V characteristics of an IZTO/β-Ga_2_O_3_ Schottky barrier diode was simulated by SILVACO-Atlas and compared with measurements. The effects of the IZTO workfunction and the interfacial layer electron affinity and trap concentrations were studied for further agreement with measurements made in the dark. Then, we demonstrated the IZTO/β-Ga_2_O_3_ self-powered Schottky photodetector with a high photo-to-dark ratio and responsivity of $3.70\times 10^{5}$ and 0.64 (mA/W) under 255 nm illumination with 1 mW/cm^2^ for 0 V, respectively. Finally, with increasing power density, the photocurrent increased, and a 17.80 mA/W responsivity under 10 mW/cm^2^ was obtained.

## Figures and Tables

**Table 1:** Properties of each layer of the studied SBD [2,3,23].

Parameters	Sn: β-Ga_2_O_3_	Si: β-Ga_2_O_3_
Bandgap (eV)	4.8	4.8
Affinity (eV)	4	4
$\mathrm{Hole}\;\mathrm{mobility}\;({\mathrm{cm}}^{2}V^{-1}s^{-1})$	10	10
$\mathrm{Electron}\;\mathrm{mobility}\;({\mathrm{cm}}^{2}V^{-1}s^{-1})$	172	300
$m_{e}^{*}/m_{0}$	0.28	0.28
$m_{h}^{*}/m_{0}$	0.35	0.35
Relative permittivity	12.6	11
$N_{c}({\mathrm{cm}}^{-3})$	$3.7\times 10^{18}$	$3.7\times 10^{18}$
$N_{v}({\mathrm{cm}}^{-3})$	$5\times 10^{18}$	$5\times 10^{18}$
$N_{d}({\mathrm{cm}}^{-3})$	$1\times 10^{18}$	$3\times 10^{16}$
Minority carrier diffusion length (nm)	450	450
Saturation velocity (cm s^⁻1^)	$10^{7}$	$10^{7}$

**Table 2:** Characteristics of the Sn-doped and Si-doped β-Ga_2_O_3_ traps considered in this work [1,2,3,24].

Traps	Trap Level $\left(E_{c}-E)\;(\mathrm{eV}\right)$	Concentration $\left(cm^{-3}\right)$	$\mathrm{Capture}\;\mathrm{Cross}\;\mathrm{Sec}\mathrm{tion}\;\sigma_{n}\;({\mathrm{cm}}^{2})$	$\sigma_{n}$ $/\sigma_{p}$
**Sn-doped β-Ga_2_O_3_ Bulk layer**	0.55	$3\times 10^{13}$	$2\times 10^{-14}$	100
	0.74	$2\times 10^{16}$	$2\times 10^{-14}$	100
	1.04	$4\times 10^{16}$	$2\times 10^{-14}$	10
**Si-doped β-Ga_2_O_3_ thin layer**	0.60	$3.6\times 10^{13}$	$2\times 10^{-14}$	100
	0.75	$4.6\times 10^{13}$	$2\times 10^{-14}$	100
	0.72	$4.6\times 10^{13}$	$2\times 10^{-14}$	100
	1.05	$1.1\times 10^{14}$	$2\times 10^{-14}$	10

**Table 3:** Output parameters from simulation and measurement.

Parameter	n	$\phi_{B}$ (eV)	$R_{s}$ $\left(Ω\;{\mathrm{cm}}^{2}\right)$	$R_{on}$ (Ω cm^2^)	$J_{s}\;(A/{\mathrm{cm}}^{2})$
Simulation	1.02	1.25	1.78	1.01	$1.72\times 10^{-12}$
Measurement	1.03	1.29	1.91	1.04	$1.11\times 10^{-11}$

## Data Availability

Data presented in this article is available on request from the corresponding author.

## References

[1] Galazka Z. (2018). β-Ga_2_O_3_ for wide-bandgap electronics and optoelectronics. Semicond. Sci. Technol..

[2] Labed M., Sengouga N., Labed M., Meftah A., Kyoung S., Kim H., Rim Y.S. (2021). Modeling a Ni/β-Ga_2_O_3_ Schottky barrier diode deposited by confined magnetic-field-based sputtering. J. Phys. D Appl. Phys..

[3] Labed M., Sengouga N., Labed M., Meftah A., Kyoung S., Kim H., Rim Y.S. (2021). Modeling and analyzing temperature-dependent parameters of Ni/β-Ga_2_O_3_ Schottky barrier diode deposited by confined magnetic field-based sputtering. Semicond. Sci. Technol..

[4] Labed M., Sengouga N., Meftah A., Labed M., Kyoung S., Kim H., Rim Y.S. (2020). Leakage Current Modelling and Optimization of β-Ga_2_O_3_ Schottky Barrier Diode with Ni Contact under High Reverse Voltage. ECS J. Solid State Sci. Technol..

[5] Pearton S.J., Ren F., Tadjer M., Kim J. (2018). Perspective: Ga_2_O_3_ for ultra-high power rectifiers and MOSFETS. J. Appl. Phys..

[6] Thomas S.R., Adamopoulos G., Lin Y.-H., Faber H., Sygellou L., Stratakis E., Pliatsikas N., Patsalas P.A., Anthopoulos T.D. (2014). High electron mobility thin-film transistors based on Ga_2_O_3_ grown by atmospheric ultrasonic spray pyrolysis at low temperatures. Appl. Phys. Lett..

[7] Grillo A., Barrat J., Galazka Z., Passacantando M., Giubileo F., Iemmo L., Luongo G., Urban F., Dubourdieu C., Di Bartolomeo A. (2019). High field-emission current density from β-Ga_2_O_3_ nanopillars. Appl. Phys. Lett..

[8] Jian G., He Q., Mu W., Fu B., Dong H., Qin Y., Zhang Y., Xue H., Long S., Jia Z. (2018). Characterization of the inhomogeneous barrier distribution in a Pt/(100)β-Ga_2_O_3_ Schottky diode via its temperature-dependent electrical properties. AIP Adv..

[9] Labed M., Park J.H., Meftah A., Sengouga N., Hong J.Y., Jung Y.-K., Rim Y.S. (2021). Low Temperature Modeling of Ni/β-Ga_2_O_3_ Schottky Barrier Diode Interface. ACS Appl. Electron. Mater..

[10] Peng B., Yuan L., Zhang H., Cheng H., Zhang S., Zhang Y., Zhang Y., Jia R. (2021). Fast-response self-powered solar-blind photodetector based on Pt/β-Ga_2_O_3_ Schottky barrier diodes. Optik.

[11] Kim H., Seok H.-J., Park J.H., Chung K.-B., Kyoung S., Kim H.-K., Rim Y.S. (2021). Fully Transparent InZnSnO/β-Ga_2_O_3_/InSnO Solar-Blind Photodetectors with High Schottky Barrier Height and Low-Defect Interfaces. J. Alloys Compd..

[12] Guo D., Liu H., Li P., Wu Z., Wang S., Cui C., Li C., Tang W. (2017). Zero-Power-Consumption Solar-Blind Photodetector Based on β-Ga_2_O_3_/NSTO Heterojunction. ACS Appl. Mater. Interfaces.

[13] Qin Y., Li L., Zhao X., Tompa G.S., Dong H., Jian G., He Q., Tan P., Hou X., Zhang Z. (2020). Metal–Semiconductor–Metal ε-Ga_2_O_3_ Solar-Blind Photodetectors with a Record-High Responsivity Rejection Ratio and Their Gain Mechanism. ACS Photonics.

[14] Zhuo R., Wu D., Wang Y., Wu E., Jia C., Shi Z., Xu T., Tian Y., Li X. (2018). A self-powered solar-blind photodetector based on a MoS_2_/β-Ga_2_O_3_ heterojunction. J. Mater. Chem. C.

[15] Liu Z., Wang X., Liu Y., Guo D., Li S., Yan Z., Tan C.-K., Li W., Li P., Tang W. (2019). A high-performance ultraviolet solar-blind photodetector based on a β-Ga_2_O_3_ Schottky photodiode. J. Mater. Chem. C.

[16] Zou Y., Zhang Y., Hu Y., Gu H. (2018). Ultraviolet Detectors Based on Wide Bandgap Semiconductor Nanowire: A Review. Sensors.

[17] Chen Y., Zhang K., Yang X., Chen X., Sun J., Zhao Q., Li K., Shan C. (2020). Solar-blind photodetectors based on MXenes–β-Ga_2_O_3_ Schottky junctions. J. Phys. D Appl. Phys..

[18] Chen X., Liu K., Zhang Z., Wang C., Li B., Zhao H., Zhao D., Shen D. (2016). Self-Powered Solar-Blind Photodetector with Fast Response Based on Au/β-Ga_2_O_3_ Nanowires Array Film Schottky Junction. ACS Appl. Mater. Interfaces.

[19] Zhi Y., Liu Z., Chu X., Li S., Yan Z., Wang X., Huang Y., Wang J., Wu Z., Guo D. (2020). Self-Powered β-Ga2O3 Solar-Blind Photodetector Based on the Planar Au/Ga_2_O_3_ Schottky Junction. ECS J. Solid State Sci. Technol..

[20] Liu Z., Zhi Y., Zhang S., Li S., Yan Z., Gao A., Zhang S., Guo D., Wang J., Wu Z. (2021). Ultrahigh-performance planar β-Ga_2_O_3_ solar-blind Schottky photodiode detectors. Sci. China Technol. Sci..

[21] Cui S., Mei Z., Zhang Y., Liang H., Du X. (2017). Room-Temperature Fabricated Amorphous Ga_2_O_3_ High-Response-Speed Solar-Blind Photodetector on Rigid and Flexible Substrates. Adv. Opt. Mater..

[22] Labed M., Sengouga N., Meftah A., Meftah A., Rim Y.S. (2021). Study on the improvement of the open-circuit voltage of NiOx/Si heterojunction solar cell. Opt. Mater..

[23] Polyakov A.Y., Lee I.-H., Smirnov N.B., Yakimov E.B., Shchemerov I.V., Chernykh A.V., Kochkova A.I., Vasilev A.A., Carey P.H., Ren F. (2019). Defects at the surface of β-Ga_2_O_3_ produced by Ar plasma exposure. APL Mater..

[24] Labed M., Sengouga N., Rim Y.S. (2022). Control of Ni/β-Ga_2_O_3_ Vertical Schottky Diode Output Parameters at Forward Bias by Insertion of a Graphene Layer. Nanomaterials.

[25] Sze S.M., Ng K.K. (2006). Physics and Properties of Semiconductors—A Review. Phys. Semicond. Devices.

[26] Carey P.H., Yang J., Ren F., Hays D.C., Pearton S.J., Kuramata A., Kravchenko I.I. (2017). Improvement of Ohmic contacts on Ga_2_O_3_ through use of ITO-interlayers. J. Vac. Sci. Technol. B.

[27] Oshima T., Wakabayashi R., Hattori M., Hashiguchi A., Kawano N., Sasaki K., Masui T., Kuramata A., Yamakoshi S., Yoshimatsu K. (2016). Formation of indium–tin oxide ohmic contacts for β-Ga_2_O_3_. Jpn. J. Appl. Phys..

[28] Li K.-D., Chen P.-W., Chang K.-S., Hsu S.-C., Jan D.-J. (2018). Indium-Zinc-Tin-Oxide Film Prepared by Reactive Magnetron Sputtering for Electrochromic Applications. Materials.

[29] Hakkoum H., Tibermacine T., Sengouga N., Belahssen O., Ghougali M., Benhaya A., Moumen A., Comini E. (2020). Effect of the source solution quantity on optical characteristics of ZnO and NiO thin films grown by spray pyrolysis for the design NiO/ZnO photodetectors. Opt. Mater..

[30] Hassanien A.S., Akl A.A. (2015). Influence of composition on optical and dispersion parameters of thermally evaporated non-crystalline Cd_50_S_50−x_Se_x_ thin films. J. Alloys Compd..

[31] Yang W.-C., Rodriguez B.J., Gruverman A., Nemanich R.J. (2004). Polarization-dependent electron affinity of LiNbO_3_ surfaces. Appl. Phys. Lett..

[32] Mohamed M., Irmscher K., Janowitz C., Galazka Z., Manzke R., Fornari R. (2012). Schottky barrier height of Au on the transparent semiconducting oxide β-Ga_2_O_3_. Appl. Phys. Lett..

[33] Lee H.Y., Lichtenwalner D.J., Jur J.S., Kingon A.I. (2019). Investigation of Conducting Oxide and Metal Electrode Work Functions on Lanthanum Silicate High-k Dielectric. ECS Trans..

[34] Choi K.-H., Nam H.-J., Jeong J.-A., Cho S.-W., Kim H.-K., Kang J.-W., Kim D.-G., Cho W.-J. (2008). Highly flexible and transparent InZnSnO_x_∕Ag∕InZnSnO_x_ multilayer electrode for flexible organic light emitting diodes. Appl. Phys. Lett..

[35] Buchholz D.B., Proffit D.E., Wisser M.D., Mason T.O., Chang R.P.H. (2012). Electrical and band-gap properties of amorphous zinc–indium–tin oxide thin films. Prog. Nat. Sci. Mater. Int..

[36] Sato K., Yasumura Y. (1985). Study of forward I-V plot for Schottky diodes with high series resistance. J. Appl. Phys..

[37] Hu Q., Wang P., Yin J., Liu Y., Lv B., Zhu J.-L., Dong Z., Zhang W., Ma W., Sun J. (2020). High-Responsivity Photodetector Based on a Suspended Monolayer Graphene/RbAg_4_I_5_ Composite Nanostructure. ACS Appl. Mater. Interfaces.

[38] Periyanagounder D., Gnanasekar P., Varadhan P., He J.-H., Kulandaivel J. (2018). High performance, self-powered photodetectors based on a graphene/silicon Schottky junction diode. J. Mater. Chem. C.

[39] Wu D., Zhao Z., Lu W., Rogée L., Zeng L., Lin P., Shi Z., Tian Y., Li X., Tsang Y.H. (2021). Highly sensitive solar-blind deep ultraviolet photodetector based on graphene/PtSe2/β-Ga_2_O_3_ 2D/3D Schottky junction with ultrafast speed. Nano Res..

